# Editorial: Emotions and artificial intelligence

**DOI:** 10.3389/fpsyg.2025.1720713

**Published:** 2025-12-15

**Authors:** Simone Belli, Angel Barrasa, Cristian López Raventós, Mariacarla Marti

**Affiliations:** 1 Complutense University of Madrid, Madrid, Spain; 2 Universidad Ecotec, Samborondon, Ecuador; 3 Universidad de Zaragoza, Zaragoza, Spain; 4 Universidad Nacional Autonoma de Mexico Escuela Nacional de Estudios Superiores Unidad Morelia, Morelia, Mexico

**Keywords:** emotions, artificial intelligence, social psychology, ChatGPT, music

In 2023, we launched this Research Topic for *Frontiers in Psychology* to explore the intersection between emotions and Artificial Intelligence (AI). That year marked a turning point, the impact of generative AI expanded rapidly, influencing nearly every aspect of our lives. This profound transformation prompted us to open a dedicated space for reflection and scientific dialogue. At the time, there was a notable gap in the scientific literature regarding the psychological dimensions of AI, particularly how emotions are shaped by, interpreted through, or integrated into artificial systems. Our aim was to address this gap and encourage interdisciplinary contributions bridging psychology, emotion studies, and AI technologies.

Over the course of this initiative, we received numerous valuable submissions. After careful review, we selected 15 contributions that we believe best represent the current advancements and perspectives in this emerging field. These works significantly enhance our understanding of the emotional dimensions of human-AI interaction, and we are proud to present them in this Research Topic.

In the paper titled “*Social and ethical impact of emotional AI advancement: the rise of pseudo-intimacy relationships and challenges in human interactions*” (Wu), author argues that the integration of emotional intelligence into algorithmic platforms is ushering in a new human interaction model: the pseudo-intimacy relationship. The goal of the work is to theoretically define this pseudo-intimacy and conclude that, despite the tensions and contradictions EAI introduces into the social environment, its technological progress can and should continue, provided that its profound impact on existing social paradigms and its ethical challenges are fully addressed.

The paper, “*Intrinsic motivation in cognitive architecture: intellectual curiosity originated from pattern discovery*” (Nagashima et al.), proposes a novel mechanism for intrinsic motivation rooted in the perspective of human cognition, specifically defining intellectual curiosity as the drive to discover novel, compressible patterns in data. Through simulations involving three ACT-R models with varying levels of thinking navigating different mazes, the study found that increasing intellectual curiosity negatively impacted task completion in models with lower thinking levels but positively affected those with higher thinking levels.

The article, “*The role of socio-emotional attributes in enhancing human-AI collaboration*” (Kolomaznik et al.), investigates how incorporating socio-emotional attributes like trust, empathy, and rapport can significantly optimize human-AI interactions and boost productivity. The analysis suggests that when AI is designed to align with human emotional and cognitive needs, it fosters deeper trust and empathetic understanding, leading to marked improvements in collaborative efficiency, productivity, and the ethical integrity of human-AI relationships.

The exploratory study, “*Emotion topology: extracting fundamental components of emotions from text using word embeddings*” (Plisiecki and Sobieszek), investigates the potential of word embeddings as a novel, data-driven method for emotion decomposition analysis. The study concludes that word embeddings are a promising, theory-agnostic tool for uncovering emotional nuances and suggests that this methodology could be broadly applied to enrich the understanding of emotional and other psychological constructs in an ecologically valid way.

The article “*Emotional responses of Korean and Chinese women to Hangul phonemes to the gender of an artificial intelligence voice*” (Lee et al.) investigates how cultural background and AI voice gender influence emotional responses to phonemes. The findings demonstrate that even phonemic units without semantic meaning can elicit varying emotional responses depending on both cultural context and AI voice gender.

The article “*Impact of media dependence: how emotional interactions between users and chat robots affect human socialization?*” (Yuan et al.) explores how media dependence shapes emotional interactions between users and chatbots, focusing on the Replika platform. Results indicate that while most users are light users who invest moderate time and emotions, chatbot interactions provide satisfaction, companionship, and relief from loneliness.

The article “*Is it possible for people to develop a sense of empathy toward humanoid robots and establish meaningful relationships with them?*” (Morgante et al.) presents a systematic review on empathy in human–robot interaction (HRI). Findings suggest that empathy is more likely when robots display anthropomorphic traits, such as facial expressions, gestures, or emotional narratives. The authors conclude that further research is needed to refine empathy models in robotics while ensuring responsible and beneficial applications for society.

The article “*A research on copyright issues impacting artists emotional states in the framework of artificial intelligence*” (Kambur and Dolunay) examines how copyright issues, particularly in the context of AI-generated art, impact artists' emotional states and creative motivation. The research emphasizes the importance of updating national and international copyright laws to address digital and AI-related works to better protect artists' rights and emotional wellbeing. The study advocates for increased awareness and educational activities to raise understanding of copyright issues.

In “*Implementing machine learning techniques for continuous emotion prediction from uniformly segmented voice recordings*,” Diemerling et al. introduce an innovative approach using neural networks to detect emotions in short voice segments in real time, demonstrating promising results for enhancing human-machine interaction.

Dolunay and Temel, in “*The relationship between personal and professional goals and emotional state in academia*,” examine how academics' aspirations and emotions influence the ethical use of AI, emphasizing the need for training programs and institutional ethics to prevent unethical conduct.

Tretter, in “*Equipping AI-decision-support-systems with emotional capabilities? Ethical perspectives*,” discusses the ethical implications of endowing decision-support systems with emotional capacities, advocating for a balance between potential benefits and risks of manipulation and accountability loss.

Vistorte et al., in “*Integrating artificial intelligence to assess emotions in learning environments*,” conduct a systematic review of the current state of AI in emotional assessment within education, highlighting its potential to personalize learning but warning of challenges related to accuracy, privacy, and ethics. Together, these studies demonstrate the importance of advancing the use of emotional AI responsibly, combining technological innovation with deep ethical and social reflection.

The study, “*Decoding emotional responses to AI-generated architectural imagery*” by Zhang et al., investigated how AI-generated images evoke emotion and whether an architectural background influences that perception. The results showed that AI is effective at conveying positive emotions, particularly joy in interior settings, but struggles with negative ones.

The study “*Artificial intelligence and social intelligence: preliminary comparison study between AI models and psychologists*” by Sufyan et al. examined the social intelligence (SI) of three AI models against that of 180 psychology students and doctoral candidates. The study concludes that AI's ability to understand emotions and social behavior is developing at a rapid pace and suggests that these models could become valuable tools in counseling and psychotherapy.

A study by Kumar and Kathiravan titled “*Emotion recognition from MIDI musical file using Enhanced Residual Gated Recurrent Unit architecture*” investigates a new method for detecting emotions in music. The researchers used a sophisticated AI model called the Enhanced Residual Gated Recurrent Unit (RGRU) to analyze MIDI music files. The findings highlight the potential for using this technology to create more advanced music recommendation systems.

The 15 contributions gathered in this Research Topic successfully addressed the initial scientific gap, offering a rich, interdisciplinary perspective on the intersection of emotions and Artificial Intelligence at a pivotal time of rapid generative AI expansion. Collectively, these works reveal a dual imperative: to foster technological advancement in emotional AI while rigorously upholding ethical and human-centric principles. The Research Topic establishes that AI is fundamentally reshaping psychological and social paradigms. For instance, the concept of the pseudo-intimacy relationship highlights the need to understand how human emotional needs are being met, and potentially substituted, by AI, urging developers and policymakers to address risks like human alienation proactively. Simultaneously, research into intrinsic motivation shows that sophisticated cognitive architectures can model complex human drives, suggesting that future AI innovation is closely tied to designing systems that genuinely emulate higher-level thinking, curiosity, and goal-directed intention. Furthermore, the findings on the enhanced social intelligence of advanced AI models compared to human psychologists underscore AI's rapid development in simulating human social cognition, pointing to its growing role as a potential tool in fields like counseling and psychotherapy. A central theme across the articles is the necessity of a holistic, human-centric approach to AI development. Studies consistently advocate for incorporating socio-emotional attributes like trust and empathy into AI design to optimize collaboration and productivity. However, this progress is tempered by ethical warnings: the review on empathy in human-robot interaction raises concerns about emotional manipulation, while papers on decision-support systems and academic ethics stress the dangers of accountability loss and unethical conduct. Crucially, the research on copyright issues underscores that legal and institutional frameworks must adapt to protect artists' emotional wellbeing and creative motivation in the face of AI-generated content. These findings collectively demonstrate that advancing emotional AI responsibly requires balancing technological innovation with robust ethical reflection and public literacy. Moreover, the research significantly advances the methodology for understanding and measuring emotion; the validation of word embeddings for emotion decomposition analysis provides a novel, data-driven tool for psychological research. Simultaneously, studies focusing on specific applications, such as continuous emotion prediction from voice, emotion recognition in musical files, and the decoding of emotional responses to AI-generated architectural imagery, showcase the practical potential of these technologies across diverse industries. Critically, the identification of significant cross-cultural differences in emotional responses to AI voice gender emphasizes that future AI applications must prioritize cultural nuance and customization to achieve truly optimized and effective human-machine interaction. This body of work provides a solid foundation, calling for continued research to refine models of empathy and motivation, establish clearlegal frameworks, and ensure that AI's expanding emotional capabilities are deployed for the beneficial and ethical advancement of society.

